# The Views of Adult Weight Management Dietitians on Service Provision for People With Obesity and Severe Mental Illness and/or Learning Disability: A Qualitative Study

**DOI:** 10.1111/nbu.70013

**Published:** 2025-06-05

**Authors:** Anita Attala, Amelia A. Lake, Emma L. Giles

**Affiliations:** ^1^ Nutrition and Dietetics Department Northumbria Healthcare NHS Foundation Trust Newcastle upon Tyne UK; ^2^ School of Health and Life Sciences Teesside University Middlesbrough UK; ^3^ Fuse‐ The Centre for Transactional Research in Public Health Newcastle UK

**Keywords:** dietitian, learning disability, obesity, qualitative, severe mental illness, specialist adult weight management, tier 3

## Abstract

Specialist adult weight management (AWM) services (tier 3) provide multidisciplinary support for people with obesity to manage their weight. Many people with severe mental illness (SMI)/learning disability also have obesity. This study explored the opinions of specialist AWM dietitians in the North‐East and North Cumbria (NENC) region regarding their skills, knowledge, and services for supporting people with obesity and SMI/learning disability. Dietitians (*n* = 9) were purposively selected and interviewed using Microsoft Teams in July 2023. The data was thematically analysed. Dietetic pre‐registration training on SMI/learning disability was inconsistent. Dietitians' confidence in supporting people with SMI/learning disability was wide‐ranging. Six themes were identified: training, resources, service provision, networking & external influences, assessment and compassion & self‐efficacy. Specialist AWM dietitians in the NENC region are compassionate and want to provide a quality service for people with obesity and SMI/learning disability. However, they sometimes feel they fail these service users through a lack of training and resources. Training, accessible resources, and collaboration with mental health dietitians may improve AWM dietitians' confidence and skills when supporting people with SMI/learning disability and may reduce stigma. The British Dietetic Association (BDA) may wish to ensure training on SMI and learning disability is available for all dietitians, along with resource sharing. Additionally, limited staffing and waiting lists may impact the provision of reasonable adjustments required for people with additional needs. Alternative pathways for people with obesity and additional requirements may be of benefit. More comprehensively completed referrals and additional funding may facilitate this.

## 1 Introduction

The United Kingdom (UK) has four levels of adult weight management (AWM) services, referred to as ‘tiers’ (Public Health England 2017). Tier 3 specialist AWM services have a multidisciplinary team, which may include a physician, psychologist, specialist dietitian, specialist nurse and physiotherapist/exercise specialist (NHS England 2014). The North‐East and North Cumbria (NENC) region has six NHS Trusts, which offer specialist AWM services (Coxon 2023). This study explored the opinions of dietitians working in tier 3 specialist AWM services in the (NENC) region.

People with a learning disability, who have a substantially reduced capability to understand new/complex information or live independently (Public Health England 2023), and individuals with a severe mental illness (SMI) (e.g., schizophrenia, psychoses, bipolar disorder or personality disorders) have a higher risk of living with overweight/obesity (Robertson et al. 2021; Bradshaw and Mairs 2014), which may be further compounded by medication (Brown et al. 2010; NHS England 2019). Furthermore, people with SMI/learning disability are at risk of dying 15–25 years earlier than the general population, often from obesity‐related diseases (Robertson et al. 2021; Bradshaw and Mairs 2014). Indeed, health inequalities are well documented for people with SMI/learning disability, particularly around accessing a healthy diet and public services to help with weight management (The Kings' Fund 2024; Gov.uk 2022).

Dietitians have little pre‐registration training on SMI or learning disability (Sloan and Frascina 2023; Ash et al. 2008; Shakespeare et al. 2009). Despite this being reported for many years, at the time of writing, the British Dietetic Association's (BDA) pre‐registration framework does not specify SMI/learning disability as areas dietitians should be taught (British Dietetic Association 2020). Furthermore, dietitians report a lack of training to conduct capacity assessments on diet/health with patients with a learning disability (Sloan and Frascina 2023).

Additionally, the Health and Care Professions Council (HCPC), the regulatory body for dietitians, stipulates several standards of proficiency, which relate to the dietitians' skills and knowledge required to support people with SMI/learning disability (Health and Care Professions Council 2023). However, lack of training may lead to a potential gap in dietitians' knowledge/skills when supporting people with SMI/learning disability (Sloan and Frascina 2023; Ash et al. 2008; Shakespeare et al. 2009), making it difficult for dietitians to confidently and competently adhere to these standards (Caffrey 2023).

There is limited literature on the knowledge/skills of dietitians for supporting people with SMI/learning disability. Where evidence exists, dietitians are reported to have limited training in mental health and therefore lack the skills to support people with SMI/learning disability (Ash et al. 2008; Shakespeare et al. 2009). Furthermore, other healthcare professions also identify limited training and understanding for supporting people with SMI/learning disability, which may lead to poor patient outcomes and discrimination against this client group and therefore widen the health inequality gap in this population (Shakespeare et al. 2009; Alwar and Addis 2022; Giandinoto and Edward 2015; Ho et al. 2022).

Moreover, the Equality Act (2010) recommends that reasonable adjustments are made for people with a disability (Government Equalities Office 2011). It is, therefore, important that services know if a patient has a disability, so they can be suitably supported (George et al. 2022). Yet, some hospitals do not routinely provide, audit or monitor the reasonable adjustments provided for people with SMI/learning disability, thus adding to the health inequality gap (Heslop et al. 2018).

There are no national referral criteria for AWM services as they are locally commissioned by Integrated Care Boards (Royal College of Surgeons of England 2014; Coxon 2023). However, National Institute for Health and Care Excellence (NICE) suggests referring a person to specialist tier 3 AWM if they have obesity (BMI > 30 kg/m^2^) along with ‘complex disease states or needs that cannot be managed adequately in tier 2 (for example, the additional support needs of people with learning disabilities)’ or when ‘conventional treatment has been unsuccessful’ (Public Health England 2017, 64). It is, therefore, likely that specialist AWM dietitians require the skills and knowledge to support patients with SMI/learning disability.

There is a lack of literature regarding the perceived knowledge/skills and service provision of specialist AWM dietitians for supporting people with obesity and SMI and/or learning disability. By using semi‐structured interviews with specialist AWM dietitians in the NENC region, this study aimed to explore: (i) if specialist AWM dietitians feel they have the appropriate skills/knowledge to care for people with SMI/learning disability, (ii) what might enhance their skills/knowledge, (iii) whether specialist AWM services have the systems in place to care for people with SMI/learning disability and (iv) what might enhance their care. To our knowledge, this is the first study of its kind.

## 2 Methods

A phenomenological approach was applied using semi‐structured interviews to explore the views and experiences of specialist AWM dietitians (Tong et al. 2007). To ensure appropriate study design and reporting, the consolidating criteria for reporting qualitative research (COREQ) tool was used (Tong et al. 2007).

Ethical approval was obtained by Teesside University and via the Integrated Research Application System (IRAS: 324599). The study gained Caldicott approval and sponsorship from Northumbria Healthcare NHS Foundation Trust. Written consent was obtained prior to the interview.

The semi‐structured interview was piloted (Majid et al. 2017; Aung et al. 2021) with a dietitian who previously worked in a specialist AWM service in the region. The interview was subsequently adapted by adding a question to explore the dietitian's experience around assessing capacity for diet/weight.

All NHS Trusts in the NENC region with a specialist AWM service were sent an invitation to participate. The dietetics/AWM service lead of each unit was requested to forward an invitation email with the participant information sheet to the relevant dietitians. The dietitians were invited to contact the interviewer (AA) directly to express an interest in participating and arrange a mutually suitable interview time.

The interviews were conducted using Microsoft Teams in July 2023 (Khan and MacEachen 2022; Braun et al. 2017). Participants were interviewed once, and transcripts were not returned to participants for review (Birt et al. 2016); however, participants had access to their video recording until transcription was completed.

To allow a relevant clinical discussion on how the dietitian might support an individual with obesity, SMI and mild learning disability, part of the semi‐structured interview invited the dietitians to consider a case study, which was developed for the study, or a patient under their care (Attala et al. 2023). The dietitians were asked to rate their confidence in supporting the patient, whereby 1 = no confidence and 10 = highly confident. The reported rating is referred to as their ‘confidence score’.

The interviews were recorded and initially transcribed by Microsoft Teams; subsequently transcribed ad verbatim by the interviewer (AA). The recordings, which only the participant and interviewer (AA) could view, were deleted after transcription. Analysis and coding of the transcriptions were performed using a single coder: the interviewer (AA) (Keene 2023).

This study was undertaken as part of a Clinical Research MRes. The interviewer (AA) is an experienced, female dietitian, working as the Lead Dietitian in a specialist AWM service in the North‐East of England. The participants were informed of this in the participant information sheet. To improve study trustworthiness, the questions were open and brief fieldnotes were kept reflecting upon what went well/did not in the interview (Tessier 2012). AA has previous experience of conducting semi‐structured interviews and was supervised by a wider research team experienced in qualitative methods, thus ensuring reliability (Pizzolato and Dierickx 2023).

Descriptive statistics were used to outline participants' experience. The interview transcripts were transcribed ad verbatim, analysed, and coded by AA both manually and using Nvivo (Lumivero NVivo Pro 12, 2019 www.lumivero.com) (Braun and Clarke 2022). Thematic analysis was used to analyse the data (Braun et al. 2017).

## 3 Results

Dietitians from five of the six possible NHS Trusts responded to participate. The interviews lasted an average of 49 min (range: 37–65 min). Most participants were in a quiet room on their own. Two participants (P6 and P8) took part while in shared offices; however, they used headphones, were able to speak freely and were not interrupted.

A total of eleven dietitians expressed an interest in participating. Using purposeful sampling (Silverman 2010; Guest et al. 2006) to ensure a range of genders, dietetic experience, experience of working in AWM and NHS Trusts, a total of nine dietitians were interviewed. Two dietitians were not selected as it would have over‐represented a Trust with an integrated tier3/tier 4 service, which is only available for those who want to be considered for bariatric surgery (Smith 2024). To ensure anonymity, no further information can be given about this Trust (Dougherty 2021). The interviews proceeded until no further themes emerged; thus, data saturation was perceived to have been achieved (Guest et al. 2006; Braun and Clarke 2021).

Many of the dietitians interviewed were experienced and had worked in AWM services for some time. Most of the dietitians worked part‐time (0.2–0.9 whole time equivalent) in the specialist AWM service (Table 1), with other roles in bariatrics, diabetes and care homes.

**TABLE 1:** Dietitians' overall experience; experience of working in adult weight management and current workload in adult weight management.

	Number and percentage of dietitians	
Years qualified as dietitian		
0–4 years	*n* = 2	22%
5–10 years	*n* = 2	22%
11 years or more	*n* = 5	56%
Years working in adult weight management		
0–4 years	*n* = 4	45%
5–10 years	*n* = 3	33%
11 years or more	*n* = 2	22%
Full‐time/part‐time in adult weight management		
Full‐time	*n* = 2	22%
Part‐time	*n* = 7	78%

*Note*: To ensure confidentiality gender is not reported.

### 3.1 University Training and Post‐Qualification Courses

There was a relatively even split between those qualifying from an English University (*n* = 5; 56%) and a Scottish University (*n* = 4; 44%). Two individuals, who qualified less than 4 years ago from an English University, had some taught component on SMI/learning disability on their Dietetics course. Although from the same university, the amount of input was variable, with one dietitian receiving half a module on mental health, which mainly consisted of eating disorders; the other dietitian had 2 days of external speakers whilst on placement (a day on mental health and 1 day on learning disability). They found their education on SMI/learning disability useful and transferable to their current practice.

Two dietitians, who qualified from Scottish Universities (over 5 years ago), experienced working with people with SMI/learning disability for 2–6 weeks whilst on placement. None of the dietitians who qualified in England had any placement experience of working with people with SMI or learning disability.

### 3.2 Confidence in Supporting an Individual With SMI and/or Learning Disability

Using a case study of a male with obesity, SMI and mild learning disability, participants were asked to rate their confidence to support him, whereby 1 = no confidence and 10 = highly confident. The dietitians' ‘confidence‐score’ ranged from 4 to 7.5, with an average ‘confidence‐score’ of 5.8 (Table 2). Those who gave a range of ‘confidence‐scores’, for example: 4–5, the middle score was used that is, 4.5. Two individuals lowered their ‘confidence‐ score’ during the interview, one due to lack of accessible resources for weight management: ‘confidence‐score’ dropped from 6 to 4–5; and one in relation to a discussion about capacity assessments on weight/diet: ‘confidence‐score’ dropped from 7 to 2. However, their original rating was used to calculate the average and range ‘confidence‐scores’.

**TABLE 2:** Dietitians' ‘confidence‐score’ and severe mental illness/learning disability training.

Participant	P1	P2	P3	P4	P5	P6	P7	P8	P9
‘Confidence‐scores'	4	6*	5–6	6	5	5	7.5	6–7	7 **

*Note*: Orange: University placement included severe mental illness/learning disability; Green: Taught component at university on severe mental illness/learning disability; Blue: Attended British Dietetic Association ‘Introduction to mental health’ course.

* ‘Confidence‐score’ changed from 6 to 4–5 due to lack of resources.

** ‘Confidence‐score’ changed from 7 to 2 for capacity assessment.

Participants attending the BDA's ‘Introduction to mental health course’ (blue) and experience of mental health/learning disability on student placement (orange), appear to have slightly higher ‘confidence‐scores’. However, this did not appear to be the case for those who had been taught mental health whilst at university (green) (Table 2).

Six main themes were identified from the data: training and education, resources, service provision, networking & external influences, assessment and compassion & self‐efficacy. The themes: training, resources, networking and being able to assess the individual, largely derived from the participants being asked: ‘what might help to improve your ‘confidence‐score’?’

Each theme has at least one illustrative quote. An ellipsis (…) is used for omitted words and square brackets are used for the researcher's comments to clarify the meaning/context of the quote.

#### 3.2.1 Theme 1: Training and Education

Training was perceived as the main area that could increase the dietitians' confidence to support people with SMI/learning disability. All the participants felt they lacked training and had little/no education on the subject while at university or post‐qualification.

We've not been **trained**. I don't think it was even **mentioned** at Uni (P2)

Some dietitians suggested training on SMI was important due to the link between physical and mental health. Furthermore, it was perceived that a significant proportion of patients referred to specialist AWM services have a mental health diagnosis.

…there's a lot of links….with the gut and the brain…so it makes sense for us to have training in mental health, when we know there's such a strong link between the two conditions [physical and mental health] (P1)

…that's a frightening thing, most of our patients coming through – there's ‘something’ [a mental health diagnosis], and yet our knowledge is still so lacking (P3)

Most of the dietitians were unaware of any post‐qualification training for dietitians on supporting people with SMI/learning disability.

We were having this conversation in clinic the other day ‐ about what sort of training is available and we weren't sure what there was or how to access it (P8)

Two dietitians were aware of the BDA's ‘Introduction to mental health’ course, having attended it whilst in different posts. There was a mixed reaction to the course content. One felt it was too basic, focusing too much on eating disorders. Yet, the other recalled it as an awareness session for all dietitians, not just mental health specialists.

It was quite an eating disorder focused one, there wasn't much on learning disabilities (P9)

A lot of it was around **awareness**…it was for any dietitian (P4)

Some dietitians had completed their Trust's mandatory training on learning disability. However, the lack of training on SMI was believed to contribute to poor confidence in this area.

Our Trust has just launched a mandatory training module for learning disabilities…it was brilliant. But in terms of severe mental illness no not at all. In fact, I'm even **less** confident with that (P5)

##### 3.2.1.1 Training Requirements

All participants thought training on SMI/learning disability would be useful. Most believed it would help improve their confidence in supporting people with these conditions.

I guess, a lot of what would increase my confidence is maybe a bit more training (P6)

There was a belief that *all* staff—all dietitians and any patient‐facing staff—would benefit from training on SMI/learning disability.

I think training for all staff. Not just dietitians but everyone that they [the patient with SMI/learning disability] would come across (P1)

It was thought that training might help bridge the health inequality gap for patients with SMI/learning disability and possibly reduce discrimination.

I think it [training] could be helpful. Because we are aware that…people with mental health and learning disabilities struggle to access that same quality of care and so in order to address that gap…that needs to be looked at (P9)

I mean, you could say it's relevant to everybody isn't it?…especially when we're making sure we're not discriminating against anybody (P8)

The participants offered suggestions on topics they would find helpful to be trained on, for example, mental health conditions, medication, and appropriate communication (Table 3).

**TABLE 3:** Dietitians' suggestions for training topics.

Medication—the effect medications may have on weight and on mood/energy levels/motivation
Use of appropriate terminology/how to phrase things
Capacity—how to check patient understanding and what a capacity assessment involves
Accessible resources for example, visual aids—where to access them and use them appropriately
Additional support available for the patient for example, cooking clubs
Respite available for carers
How to link and work with case workers
How the SMI/learning disability may impact a patients' life
Different types and prevalence of SMI/learning disability
Practical tips for supporting people with SMI/learning disability
Patient experiences
Other professions input/experiences

##### 3.2.1.2 Delivery of Training

It was believed that people with SMI/learning disability can be encountered in any area of dietetics, not just specialist AWM. Therefore, the dietitians felt some input on how to support people with SMI/learning disability during pre‐registration would be most beneficial.

Undergrad would be helpful… you'll come across this [supporting people with SMI/learning disability] in all settings, whether…clinical outpatients, general dietetics…so probably everybody should have [training] – not necessarily just adult weight management (P8)

think definitely as part of your degree…I think maybe placement (P1)

Some felt the BDA should be involved in either the delivery or the recommendations for delivery at pre‐registration.

…the BDA could recommend that it's done at university (P1)

The BDA could certainly do advice with regards to…the conditions, the treatment, the symptoms, the behaviours (P3)

Others felt regional training would be useful for networking and for providing ideas on local support.

I think regionally would be better because you'd know a little bit more about what services are available; about who to contact and…what the network is (P3)

Whilst training on SMI/learning disability during pre‐registration was considered beneficial, it was suggested training post‐registration would be advantageous as it might be more applicable to real life scenarios. Although, it was felt post‐qualification training should not be mandatory.

I think a lot of things…when you first learn them don't feel relevant until you're doing them…within the first couple of years of being qualified (P1)

…[training] should be encouraged and promoted rather than mandated (P5)

#### 3.2.2 Theme 2: Resources

The dietitians felt the lack of easy‐read/accessible resources was also a contributory factor for poor confidence in supporting a patient with poor literacy. When the participants were asked to rate their confidence in supporting people with SMI/learning disability, one participant lowered their ‘confidence score’ upon the realisation there was a lack of accessible resources available in AWM (‘confidence score’ reduced from 6 to 4–5).

I think I'd maybe give myself a lower score. I'd feel a bit stuck ‘cause in weight management we've not got many resources that would be suitable (P2)

It was believed that the resources used in specialist AWM services were not always appropriate for someone with poor literacy. The dietitians appeared embarrassed and uncomfortable about this.

**None** of our paperwork is adapted‐ if they get a letter through or the workbook [service resource] it's just a tonne of…information (P2)

##### 3.2.2.1 Resource Availability

Many participants were unaware of where to acquire accessible resources to support people with obesity and learning disability.

…it might be that there's…**excellent** resources somewhere that I'm not even aware of and I'm missing out on. But I don't…really know where to look (P9)

It was perceived that the BDA's mental health group has accessible resources, but they were not shared openly. Thus, making appropriate resources either inaccessible or costly for the dietitian.

I wish I had access to that group [BDA mental health group] for my patients, ‘cause I don't specifically work with people with learning disabilities, but I just wish certain things weren't behind paywalls (P6)

I could join [BDA specialist groups], but why should I have to? If there's something that they know is gonna help people, when we're being told…people with learning disabilities and mental health are being discriminated against, why should I have to pay extra money…to get resources to do our job? It should be shared openly. We shouldn't be having to pay to join a specialist group…if there's something out there that can help us all – share it openly (P9)

One dietitian had searched for accessible resources, but with little success. Another had a support worker in their service who was their Trust's learning disability link‐champion and was therefore aware of some easy‐read/accessible resources. However, they were uncomfortable about the language used in them.

There were some other resources which I thought were absolutely fantastic…specifically for learning disability – so very un‐pc terms…I think one of them was ‘So do you want to be fat?’ (P5)

##### 3.2.2.2 Resource Requirements

The dietitians gave suggestions of resources they thought might be helpful to them to support people with obesity and poor literacy, for example, visual aids, diet sheets and picture guides.

Not just diet sheets ‐ although they are helpful. I think it's more about having things to **base** our consultations around, isn't it? And picture guides and all those kind of things (P9)

It was highlighted that not only was having resources important, but also knowing where to access them and having training on how to adapt and use them was vital.

Knowing how to adapt resources (P8)

Knowing where to access resources, what resources are available, what resources are helpful to patients (P3)

#### 3.2.3 Theme 3: Service Provision

Many respondents felt their specialist AWM service was insufficiently equipped to support people with SMI and/or learning disability. Furthermore, it was perceived that poor service provision may lead to potential inequalities.

I don't think as a service we're very good at adapting things (P2)

…giving them [patients with additional needs] a separate pathway…because…we treat everybody equally, but we'll talk about health inequalities, don't we?…they obviously need different…requirements (P7)

However, despite feeling service provision and adaptation was poor, most respondents reported making some adjustments to support individuals who may require it. It was perceived that some people with a learning disability might need this. Adjustments were mainly in the form of additional clinic time, more frequent appointments, offering one‐to‐one support rather than group work, or face‐to‐face appointments rather than telephone. However, it was felt this level of support was time‐consuming and was not feasible to support large numbers and may be viewed negatively by managers, who were perceived to prefer waiting lists to be reduced.

A normal clinic appointment just is **nowhere** near enough time to go through the information and what we found is she gets really overwhelmed with too much information, so she was in clinic next week and the week after and that's kind of taking up a **lot** of time…‐ obviously we've got a lot of demand and…it feels like maybe your time's not accounted for as it would be if you were getting through the waiting list…because you're spending so much time with one patient (P1)

It was perceived that the consultation environment was also something that should be considered for some people. However, it was acknowledged that this was potentially difficult to change or control.

…making sure the setting that they feel comfortable in. But I suppose that's the only setting that we have (P8)

Our…[AWM service] see patients in a hall…I think for someone who's…over stimulated and/or…has sensory issues…it probably wouldn't be appropriate at all. I would imagine it would be quite overwhelming for them (P1)

Lack of time, poor funding, and long waiting lists were given as factors for not being able to provide more support for people with SMI/learning disability.

All of these services are so stretched (P3)

##### 3.2.3.1 Referrals to Adult Weight Management Services

Poor quality and insufficient documentation on referrals to specialist AWM services was regarded as problematic. Additionally, it was suggested some people may appear to be more ‘complex’ on paper than in real life.

It's impossible to know just based on what's written…it often just says ‘learning disability’ and you don't know what that is at all, from…are they gonna be able to walk in independently? Will they be with carers? Can they communicate?…It could be useful to have more information (P9)

Clinical triage was perceived as important. Dietitians from one Trust did not feel they had adequate triage, making it likely for a person to be insufficiently supported or provided with an unsuitable service.

…whoever triages that referral, they [should] do a bit of digging…before they get booked into a **group!** (P2)

The dietitians believed there had been an increase in referrals of individuals with a diagnosis of autistic spectrum disorder (ASD) and/or attention deficit hyperactivity disorder (ADHD) and felt ill‐equipped to support patients with these conditions.

What I am seeing a significant increase of ‐ in my own perception ‐ would be people with the ADHD diagnosis…and autism (P5)

At BOMSS [British Obesity and Metabolic Surgery Society conference], one of the lectures was on neurodivergence…and…we were like, yeah, this is becoming more and more apparent. And actually, we don't have the skills or the knowledge to be managing this (P3)

….autism's, probably one that I'm less familiar of any specific dietary advice (P9)

##### 3.2.3.2 Service Improvements

There were some suggestions for service improvements, such as creating a pathway for people with a learning disability, ensuring a consistent healthcare professional and providing training for support workers.

I think probably having a pathway that would be more of a one‐to‐one approach for them [patients with additional needs], rather than in a group setting would be…helpful. We do have the option for that, but…it's…not a definite pathway (P8)

#### 3.2.4 Theme 4: Networking and External Influences

Having a professional support network was identified as another potential means of increasing dietitians' confidence in supporting people with SMI/learning disability. Often the main sources of support were from colleagues with more experience in mental health, for example, the service psychologist(s).

We've got a very supportive psychology team and I think if it wasn't for them, then that number [confidence‐score] would have been definitely far less (P7)

It was believed support from local mental health Trusts and/or more experienced colleagues, through clinical supervision, would be useful. Dietitians working in mental health were regarded as the experts on SMI/learning disability, and the specialist AWM dietitians wanted more support and to network with them. Some specialist AWM dietitians had made links with local mental health dietitians to support specific patients, finding the dietitians working in mental health to be very supportive, providing the patient was under joint care. However, it was also viewed that there was possibly a lack of joined‐up working at times.

…clinical supervision…with other Trusts…or people who are more experienced. ‘Cause I think that in our Trust there be no point doing a clinical supervision for these patients [with SMI/learning disability], ‘cause none of us would have a clue (P1)

There's very rarely a handover between the two [acute and mental health Trusts], and I think that's hard because sometimes you feel like you're driving blind a little bit without that backing, that support (P3)

There was concern that the local mental health Trusts were going to stop seeing patients with obesity. It was felt the AWM services would consequently be inundated with referrals and ill‐equipped to support the additional patients with SMI/learning disability.

…we've heard that our local mental health Trust is turning off referrals for weight management…these patients are gonna be kind of left with no support. And then potentially coming into a service that doesn't know how to manage them (P1)

There was a sense specialist AWM services occasionally accepted referrals for individuals/conditions which ought to be excluded from the service. However, it was felt there was no alternative support/service for that person if the referral was declined. Equally, it was perceived this might not always be best for the person, and on occasion might even be detrimental.

Technically everyone with learning disabilities is outside of our referral criteria, but we make exceptions…we don't feel right; We feel that's discriminatory to say to someone…‘we can't accept you’, ‘cause they've got nothing else, so we've accepted people and…done our best (P5)

…we picked them up against our better judgement…we felt the GP didn't know what else to do with them. The patient was desperate for help and support, and they ended up, probably getting the support that was **unhelpful or detrimental**. And that's the **real** danger, isn't it? Is that because of gaps in services?…you're trying to help them because you think you're better than nothing. But sometimes, you're not better than nothing (P9)

#### 3.2.5 Theme 5: Assessment

None of the dietitians had experience of, or felt equipped to, conduct a capacity assessment for weight/diet. Nobody felt confident in this area, with one dietitian saying they would drop their ‘confidence‐score’ from 7 to a 2 if they had to think about a capacity assessment. Indeed, some dietitians had not thought of assessing capacity for diet/weight with some of their patients.

My confidence for capacity assessment would be very low, like a 2 (P9)

I'll be honest, I wouldn't think of doing that [assessing capacity] with a patient with a learning disability (P5)

There were mixed feelings about conducting capacity assessments for weight/diet. Some dietitians felt it would be useful to know how to do one, or at least have the awareness of what was involved. Others felt it was beyond their scope of practice. Some dietitians were uncomfortable about capacity assessments for diet/weight, feeling assessing capacity might be regarded as prejudiced. However, it was appreciated that assessing capacity for diet/weight was potentially an important aspect of patient care and, in some instances, safety.

if we [are] deeming that patient doesn't have capacity we're in a position where we're gonna sort of **withdraw** food to an extent ‐ for their health…I feel…quite uncomfortable with that…but it's about best interests isn't it? And I suppose if…the patient's eating themselves to a point where they're extremely unwell…then that is what needs to happen. I don't know. I don't know why I feel uncomfortable about it, to be honest. I just…it feels like a higher level of something that we should do (P4)

…we're…always so worried…about…being prejudiced against somebody, but actually sometimes it's about ‐ we've got to take it back to patient safety, haven't we? (P3)

Whilst the AWM dietitians did not perceive that they knew much about capacity assessments, many of them had an understanding of the complexity of capacity.

And there's so many steps to…capacity like someone being able to…**understand** the information and then **interpret** it and then **come to a decision** and then **communicate** that back to you (P6)

Lack of experience in conducting capacity assessments for diet/weight was regarded as the reason for poor knowledge and confidence in the area. Being able to assess capacity for diet/weight was thought to be important, and the dietitians would like to have training on it.

…**absolutely** appropriate that we should be trained on that [capacity assessments] (P5)

Despite the dietitians' belief they did not have the skills and knowledge to assess capacity for diet/weight, the majority reported they would want to assess an individual with SMI/learning disability, before deciding upon treatment. Being able to assess an individual was also viewed as something that would increase the dietitian's confidence.

…there's such a varying degree of ‐ you know there's the diagnosis on paper ‐ but it's really important to do that **assessment** yourself rather than making an assumption of what a patient may need (P8)

I think I'd feel more supported if we were able to spend more time to understand the patient before seeing them (P2)

#### 3.2.6 Theme Six: Compassion & Self‐Efficacy

All the dietitians showed compassion, wanted to do the best for the individual and to provide a good service, despite their perceived limitations. When they believed they were providing a poor service, they felt they were failing the individual and their caregivers. This made the dietitians feel they were providing sub‐standard dietetic care.

I felt like I was **absolutely** failing that patient (P2)

I felt like a…bad dietitian that day (P1)

I think it's just a confidence of knowing, you know, are we…are we doing the best for this patient? (P5)

I guess if someone looks a bit panicked [when asked to set their own goal], you feel **awful** (P6)

The dietitians described sometimes feeling ‘stuck’ and were not able to apply techniques taught during training, such as goal setting and motivational interviewing. It was perceived that these techniques may make the patient uncomfortable, but also made the dietitian feel they had not supported the patient sufficiently.

In Uni, we were always…encouraged…to get patients…setting their own goals and taking the lead. And when I first…tried that approach with…the first few patients that I had with…severe mental illness or learning disabilities…I just got a sense they felt a bit…put on the spot or ‐ it just didn't feel comfortable (P6)

I've definitely have that in clinic before, when I've felt quite stuck and…I don't know what else to suggest (P2)

It was believed weight management might not be a priority for someone experiencing a decline in mental health. Indeed, focusing on weight whilst mental health was unstable was viewed as having a possible negative impact on the individual. Additionally, dietitians thought some patients and caregivers can hold unrealistic expectations about weight loss. Dietitians found having these types of conversations difficult. The dietitians also perceived if the service provision given was incorrect/inadequate, not only could it make the dietitian feel awkward, but it could also be detrimental to the patient.

I think the main areas I've struggled with is where someone's mental health is so severe that they're not able to engage or change or that we feel…it's…**detrimental** to their mental health because…clearly ‐ it's not the priority‐ and it's not the right thing to do (P9)

I think it would be very detrimental to send them [the patient] into a workshop [a group session] and it not be suitable for them. That might just make them feel a lot worse (P4)

## 4 Discussion

This research aimed to explore the views of specialist AWM dietitians in the NENC region regarding their skills and service provision for supporting people with obesity and SMI and/or learning disability. To our knowledge, this is the first UK study focusing on non‐mental health dietitians' perspectives on this issue.

This study corroborates Australian studies, which report dietitians lack sufficient knowledge, skills, or training on mental health during pre‐registration training (Ash et al. 2008; Teasdale et al. 2023; Humphries et al. 2012). Training on SMI/learning disability varied, with some dietitians receiving education while others did not. This is likely to broaden the health inequalities gap seen in this population (FitzPatrick et al. 2021).

Dietitians reported varying levels of confidence for supporting people with SMI/learning disability, with confidence linked more to experiences than to formal training. This aligns with other studies (Buttenshaw et al. 2017; Ash et al. 2008). Despite this, all the dietitians believed additional training would enhance their skills and confidence, especially pre‐qualification and through post‐qualification updates. Such education could raise awareness, reduce discrimination and address health inequalities (Alwar and Addis 2022; Giandinoto and Edward 2015; Ho et al. 2022; FitzPatrick et al. 2021).

The dietitians expressed an interest in collaborating with mental health for training, supervision, resource sharing and support. They viewed mental health dietitians as experts in supporting people with SMI/learning disability. However, some believed mental health Trusts may not support individuals with obesity, leaving specialist AWM services supporting individuals who may not meet referral criteria and subsequently may be insufficiently supported.

Accessible, easy‐read resources for people with obesity and poor literacy were perceived as lacking, undermining the dietitians' confidence. The BDA mental health specialist group was seen as potentially having useful resources, but membership fees were seen as limiting access and contributing to health inequalities.

The dietitians were aware of the stigma and health inequalities experienced by people with SMI/learning disability (Corrigan and Watson 2002; Heslop et al. 2014; Ditchman et al. 2013); as were they aware of the weight stigma people with obesity experience (Jackson 2016; World Obesity 2022). Having a ‘double‐stigma’ for obesity and SMI/learning disability could leave the person feeling unsupported, worsening mental health and increased obesity; contributing to barriers for accessing healthcare (Mizock 2015). The dietitians were keen to avoid discrimination and reduce health inequalities.

Dietitians recognise the link between mental health and physical health, particularly around weight management (Ohrnberger et al. 2017; Centre for Mental Health 2023a). They noted the challenge of managing long‐term health conditions for people with SMI/learning disability, who require support for both their physical and mental health (Public Health England 2018; Centre for Mental Health 2023b).

The AWM dietitians reported stretched services due to limited funding, staffing and long waiting lists, a common issue across the NHS (Buse et al. 2022; NHS Confederation 2021). The dietitians believed that some services referred their patients to AWM services to reduce their own waiting lists, which sometimes led to care for people often excluded from their other services. Furthermore, whilst the specialist AWM dietitians made some reasonable adjustments to accommodate those with increased requirements, it was perceived that management would prefer waiting lists to be reduced. Other studies recommend a cultural shift is required by management to improve the care of people with additional needs, along with audits to highlight this issue (Witham et al. 2014; van Daalen et al. 2021).

The dietitians felt referrals to AWM were often incomplete, lacking details on patients' mental health or communication needs, which could impact care (Kripalani et al. 2007). Clinical triage was seen as essential for needs to be met (Johnson et al. 2018). There was also concern that some healthcare professionals pressure people with SMI/learning disability to lose weight, which may not be realistic, due to the impact of many antipsychotics (Bak et al. 2014); an issue highlighted by other authors (Teasdale et al. 2023).

There was a perception of increased referrals to specialist AWM services of people with ASD and/or ADHD. It is unclear whether this was due to an increase in diagnoses of these conditions (Underwood et al. 2022; Russell et al. 2022), or if referrals had just increased.

The dietitians felt ill‐equipped to support people with these conditions, as many individuals also have obesity (Cortese 2019; Zheng et al. 2017; Dhaliwal et al. 2019). Further research around the perceived increase in referrals of people with ASD and/or ADHD to specialist AWM would be of interest.

The recent HCPC standards for dietitians stipulate being ‘able to obtain valid consent’ and ‘importance of capacity in the context of delivering care and treatment’ (Health and Care Professions Council 2023, 2). Yet, none of the dietitians interviewed had been trained on how to assess capacity for weight/diet. Nevertheless, they gauged understanding and adapted their approach to individual needs. The authors acknowledge that since this study was completed, the BDA is now providing a course on capacity for diet, weight and health (British Dietetic Association 2023).

In conclusion, this study demonstrates that NENC region AWM dietitians are compassionate and committed to providing a good service for people with obesity and SMI/learning disability. However, their conscientiousness can lead to negative emotions they have if they believe they have failed the patient (Gordon 2022).

### 4.1 Limitations

The researcher (AA) works as the Lead AWM Dietitian for one of the participating Trusts. However, she does not line‐manage staff, but provides clinical supervision. She has worked for several years in specialist AWM in the region and is known by many of the participants. Whilst this may have helped with recruitment and open discussions, it may have led to some bias (Aburn et al. 2021; Berkovic et al. 2020). However, fieldnotes were kept highlighting/minimising these issues (Tessier 2012).

One NHS Trust did not respond, thus not all Trusts in the area were represented. A relatively small purposive sample of dietitians (*n* = 9) was selected; however, by the seventh interview, no new information was emerging; hence, data saturation was perceived to have been achieved. To comply with ethics and ensure participant anonymity, detailed information regarding the participants/participating NHS Trusts cannot be disclosed (Kaiser 2009; UK Statistics Authority 2022).

This study only explored the views of dietitians working in specialist AWM services in the NENC region. Whilst it is likely other specialist AWM services, and dietitians in other specialties, experience the same as the participants in this study, further research is required to confirm this.

The authors were unable to verify if mental health Trusts are less likely to support people with obesity; further studies are required to confirm this. Moreover, it would be of interest to obtain a patient perspective on their care and support with their obesity and SMI/learning disability.

## 5 Conclusions/Recommendations

There are clear implications for practice. Dietitians and all healthcare professionals would benefit from training to understand both physical and mental health, as often they co‐exist (Ohrnberger et al. 2017; Centre for Mental Health 2023a). This may help to improve dietitians' confidence and skills and reduce stigma for those with SMI/learning disability, thus improving patient care and narrowing the health inequality gap (Shakespeare et al. 2009; Alwar and Addis 2022; Giandinoto and Edward 2015; Ho et al. 2022; FitzPatrick et al. 2021).

Training on and the availability of accessible resources for weight management would be beneficial. Furthermore, training, supervision and linking between mental health and acute dietitians may improve clinician confidence and skills (Teasdale et al. 2023; Buttenshaw et al. 2017). The BDA may wish to take the lead on these issues: ensuring all dietitians receive education on SMI, learning disability, ADHD and ASD, particularly during pre‐registration. The BDA may wish to ensure that training post‐registration is also targeted to non‐mental health dietitians, along with sharing/developing accessible resources on obesity and networking between specialist groups.

There is a desire to support individuals with additional needs within specialist AWM services. However, whilst these services are somewhat able to adapt for those with additional needs, there is a limit to what can be done under the current conditions. Specialist AWM services are stretched and underfunded (NHS Confederation 2021). Alternative pathways for people with obesity and additional requirements may be of benefit. More comprehensively completed referrals and additional funding may facilitate this. Therefore, more funding is required to ensure parity of care.

## Disclosure

Declarations: The authors confirm they have endeavoured to ensure there is no use of combative or stigmatising language in this article. The authors affirm that this manuscript is an honest, accurate, and transparent account of the study being reported. The authors affirm that no important aspects of the study have been omitted and that any discrepancies from the study have been explained.

## Conflicts of Interest

The interviewer (Anita Attala) has a professional relationship with some of the participants involved in the study. This relationship did not influence the conduct of the interviews, data analysis, or interpretation of the results. The authors declare no other conflicts of interest.

## Data Availability

The data that support the findings of this study are available on request from the corresponding author. The data are not publicly available due to privacy or ethical restrictions.
